# The Ready to Reduce Risk (3R) Study for a Group Educational Intervention With Telephone and Text Messaging Support to Improve Medication Adherence for the Primary Prevention of Cardiovascular Disease: Protocol for a Randomized Controlled Trial

**DOI:** 10.2196/11289

**Published:** 2018-11-12

**Authors:** Jo L Byrne, Helen M Dallosso, Stephen Rogers, Laura J Gray, Ghazala Waheed, Prashanth Patel, Pankaj Gupta, Yvonne Doherty, Melanie Davies, Kamlesh Khunti

**Affiliations:** 1 Leicester Diabetes Centre University Hospitals of Leicester National Health Service Trust Leicester United Kingdom; 2 Department of Health Sciences University of Leicester Leicester United Kingdom; 3 Innovation and Research Unit Northamptonshire Healthcare Foundation Trust Northampton United Kingdom; 4 Diabetes Research Centre University of Leicester Leicester United Kingdom; 5 Department of Cardiovascular Sciences University of Leicester Leicester United Kingdom; 6 Department of Clinical Pathology and Metabolic Sciences University Hospitals of Leicester National Health Service Trust Leicester United Kingdom; 7 York Diabetes Centre York Teaching Hospital National Health Service Foundation Trust York United Kingdom

**Keywords:** medication adherence, cardiovascular diseases, primary prevention, educational intervention, telephone support, text messaging support

## Abstract

**Background:**

Poor adherence to cardiovascular medications is associated with worse clinical outcomes. Evidence for effective education interventions that address medication adherence for the primary prevention of cardiovascular disease is lacking. The Ready to Reduce Risk (3R) study aims to investigate whether a complex intervention, involving group education plus telephone and text messaging follow-up support, can improve medication adherence and reduce cardiovascular risk.

**Objective:**

This protocol paper details the design and rationale for the development of the 3R intervention and the study methods used.

**Methods:**

This is an open and pragmatic randomized controlled trial with 12 months of follow-up. We recruited participants from primary care and randomly assigned them at a 1:1 frequency, stratified by sex and age, to either a control group (usual care from a general practitioner) or an intervention group involving 2 facilitated group education sessions with telephone and text messaging follow-up support, with a theoretical underpinning and using recognized behavioral change techniques. The primary outcome was medication adherence to statins. The primary measure was an objective, novel, urine-based biochemical measure of medication adherence. We also used the 8-item Morisky Medication Adherence Scale to assess medication adherence. Secondary outcomes were changes in total cholesterol, blood pressure, high-density lipoprotein, total cholesterol to high-density lipoprotein ratio, body mass index, waist to hip ratio, waist circumference, smoking behavior, physical activity, fruit and vegetable intake, patient activation level, quality of life, health status, health and medication beliefs, and overall cardiovascular disease risk score. We also considered process outcomes relating to acceptability and feasibility of the 3R intervention.

**Results:**

We recruited 212 participants between May 2015 and March 2017. The 12-month follow-up data collection clinics were completed in April 2018, and data analysis will commence once all study data have been collected and verified.

**Conclusions:**

This study will identify a potentially clinically useful and effective educational intervention for the primary prevention of cardiovascular disease. Medication adherence to statins is being assessed using a novel urine assay as an objective measure, in conjunction with other validated measures.

**Trial Registration:**

International Standard Randomized Controlled Trial Number ISRCTN16863160; http://www.isrctn.com/ISRCTN16863160 (Archived by WebCite at http://www.webcitation.org/734PqfdQw)

**International Registered Report Identifier (IRRID):**

DERR1-10.2196/11289

## Introduction

### Background

Globally, cardiovascular disease (CVD), including heart attacks and stroke, is the leading cause of death. An estimated 17.7 million people died of CVD in 2015, representing 31% of all global deaths [1]. This is a significant burden on society. The overall CVD cost to the UK economy is approximately £19 billion [2]. However, it is estimated that 75% of all premature deaths from CVD are avoidable through effective reduction of modifiable risk factors [3].

### International Guidance on Cardiovascular Disease Risk Management for Primary Prevention

Major international guidelines for the primary prevention of CVD provide the latest evidence-based guidance on lifestyle factors (exercise, diet, smoking, weight, and alcohol) and lipid-lowering medication to reduce CVD risk [4-7]. Despite some differences in the detail of the recommendations, there is a general consensus about the benefits of exercise, the cessation of smoking, and the use of statins for people at high risk of CVD [8].

The American Heart Association recommends the prescribing of statins for the primary prevention of CVD for all patients with a serum low-density lipoprotein cholesterol level above 4.9 mmol/L, regardless of their CVD risk profile [6]. In contrast, the European Society of Cardiology is more cautious and states that statins are more frequently required by individuals with low-density lipoprotein cholesterol levels raised to above 4.9 mmol/L but may not be necessary for those with a low CVD risk score (European Society of Cardiology systematic coronary risk estimation <5%) [7]. The latest UK National Institute for Health and Care Excellence guidelines no longer use specific cholesterol targets as markers of CVD risk; instead, they advise atorvastatin 20 mg to be offered as primary prevention in patients younger than 85 years with a 10-year QRISK2 score of more than 10% [4]. The QRISK2 is the updated version of the QRISK CVD risk calculator, which was developed and validated on a UK population and addresses risk issues such as ethnicity and social deprivation [9].

In the United Kingdom, this guidance has been controversial; as such, a low threshold for starting statins would mean that a 65-year-old man would obtain a risk of 10% despite optimal body mass index, optimal cholesterol, and no comorbidities [8]. An article by Abramson et al [10] further fueled the debate over the intolerable adverse effects that are reported by 5% to 10% of patients. Consequently, in the United Kingdom, there has been a lot of negative media coverage regarding the prescribing of statins, which has resulted in many patients stopping their medication [11]. Moreover, medication adherence to CVD-preventive drugs remains a problem in both secondary and primary CVD prevention.

### Medication Adherence and Cardiovascular Disease Risk

For patients with CVD, self-reported adherence to CVD medications with a common combination of aspirin, beta-blocker, and a statin was shown to be less than 40% in both isolated and long-term follow-up surveys [12]. Moreover, despite the demonstrated safety and effectiveness of statins for CVD prevention, patient adherence to long-term statin treatment is poor [13-15]. In a recent study of a large cohort of Finnish patients (n=97,575), there was an approximately 30% increase in the risk of any cardiovascular events or death among primary prevention patients who adhered poorly to statins when they were compared with good adherers [16]. However, the evidence also suggests that the use of statins by patients is dynamic, and many patients after long periods of nonadherence will restart their treatment. This is strongly linked to clinical visits, implying that reiteration by general practitioners (GPs) of the role statins play in reducing risk may be beneficial [17].

With statin and antihypertensive treatments (the main medications used in primary CVD prevention), there are often multiple reasons for poor adherence to medication: forgetfulness, a negative attitude toward medication, frustration with poor therapeutic responses, preconceived beliefs regarding health and medication, and a poor understanding of the pros and cons of a prescribed drug. In particular, there is a lack of understanding of the benefit of CVD prevention medication and a fear of drug-related adverse events [18]. The number of barriers to medication adherence stresses the importance of how health professionals communicate risk and treatment options to patients to promote behavioral change and engage people in self-management.

Poor medication adherence is one of the key reasons why overall CVD risk remains high despite patients being prescribed statins and provided with lifestyle advice by their GPs. Therefore, it is important that patient education addresses this issue and the many misconceptions to do with statins.

### Structured Cardiovascular Disease Risk Education

Structured education has been widely advocated as a cost-effective method of promoting self-management and behavior change in individuals with chronic disease [19]. It is an alternative to one-to-one counselling and refers to group-based, patient-centered educational programs that have a clear philosophy; a written curriculum that is underpinned by appropriate learning and health behavior theories; an evidence base; and trained, quality-assessed educators [20].

In the United Kingdom, the Diabetes Education and Self Management for Ongoing and Newly Diagnosed (DESMOND) program for individuals with type 2 diabetes has demonstrated that a structured education program can be delivered within the UK National Health Service (NHS) at a national level and promote behavior change [21]. With the introduction of the NHS Health Checks and new treatment guidelines, there is a growing need for similar interventions to be developed, tested, and implemented to provide a proper pathway for the management of CVD risk.

### Study Rationale

This paper details the design and rationale of the Ready to Reduce Risk (3R) study, a randomized controlled trial to evaluate the effectiveness of a complex intervention (the 3R Education Program with follow-up short message service (SMS) text messaging and telephone support) to improve medication adherence and reduce risk in the primary prevention of CVD in high-risk individuals. We describe the development of the 3R intervention and the study methods we used so as to allow for a thorough and robust report of the methodology used to deliver and evaluate this complex intervention prior to analysis and the publication of a results paper.

## Methods

### Compliance With Standards

We used the Standard Protocol Items: Recommendations for Interventional Trials (SPIRIT) checklist (Multimedia Appendix 1) [22], in conjunction with the Consolidated Standards of Reporting Trials of Electronic and Mobile Health Applications and Online Telehealth (CONSORT-EHEALTH) checklist (Multimedia Appendix 2) [23], to describe the design of this study to ensure that this trial protocol is well reported.

### Study Design

**Figure 1:**
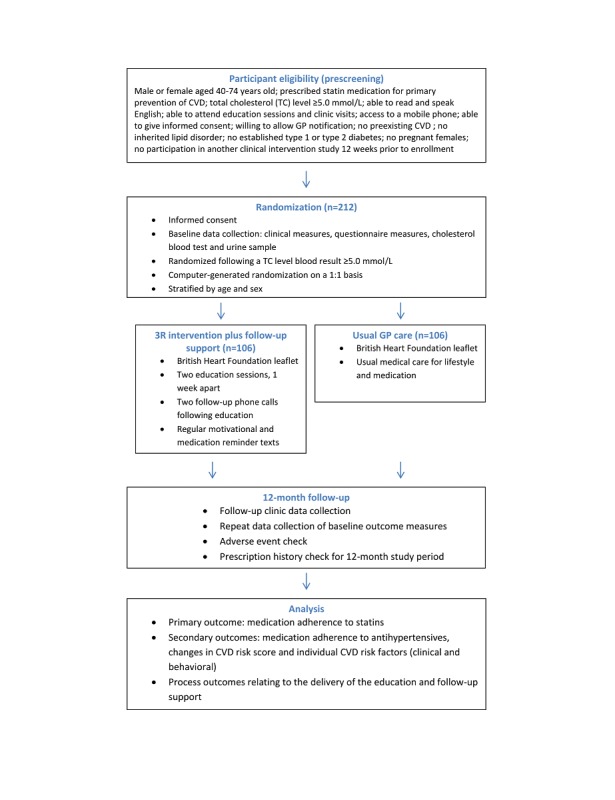
Study design. CVD: cardiovascular disease; GP: general practitioner.

The study was an open, individual, and pragmatic randomized controlled trial recruiting participants from UK general practices (attached to a single study center). We recruited patients identified as being at high risk of CVD for primary prevention (total cholesterol ≥5 mmol/L) and already prescribed statins to reduce this risk. Participants were randomly assigned to either the control group (usual GP care) or the intervention group (3R Group Education Program plus follow-up telephone and SMS text messaging support). For both the control and intervention participants, the GP was informed of a patient’s participation in the study but was not made aware of their group allocation. Both groups attended clinic visits at baseline and 12 months so that we could collect outcome data at these time points (Figure 1).

### Participants and Recruitment

#### Eligibility Criteria for Participants

Eligibility criteria were as follow: (1) male or female and aged 40-74 years, inclusive, (2) prescribed a statin medication for primary prevention of CVD that was still active, at least 12 months prior to enrollment, (3) total cholesterol level ≥5.0 mmol/L at enrollment, (4) able to speak and read English to participate effectively in the group education program, (5) willing and able to attend education sessions and clinic visits, (6) access to a mobile phone, (7) willing and able to give informed consent, (8) willing to allow their GP to be notified of participation in the study, (9) no preexisting CVD, (10) no inherited lipid disorder, (11) no established type 1 or type 2 diabetes, (12) no women who were pregnant (self-reported), and (13) no participation in another clinical intervention study in the 12 weeks prior to enrollment.

As we expected there to be participants who had repeat prescriptions for statins but were not taking them as directed, we considered a prescription to be active, for the purpose of the study, if the prescription had been issued at least twice within the previous 2 years. We recruited participants with a total cholesterol level of 5 mmol/L or greater at baseline, based on the assumption that these participants were more likely to be nonadherent to statin medication if their cholesterol levels were higher.

#### Method of Recruitment

We identified general practices from across Northamptonshire, UK to take part in the study. We developed an automatic Morbidity Query Information Export Syntax (MIQUEST) search (based on the eligibility criteria described above) for the practices to download, from a secure online site, to generate a list of potential participants to be sent invitation packs. MIQUEST is a method that is used to extract data from different types of GP database systems, using a common query language to ensure consistency. Each invitation pack contained an invitation letter, a preliminary study information leaflet, and a reply slip with prepaid envelope. Prior to mailing these packs, a clinical member of the practice staff screened the list to ensure the suitability of patients to take part.

Reply slips (from both positive and negative responders) were returned directly to the research team. We contacted all positive responders to verbally screen and confirm their eligibility for the study. Following this, eligible patients who were interested in taking part were booked to attend an initial data collection clinic. All participants were required to attend 2 data collection clinics: at baseline and at 12 months. The clinics were held in a suitable local venue, usually a community venue. Group clinics (of up to 8 participants at a time) were run by 3 trained study staff, including a qualified nurse. A full patient information sheet was sent with the clinic appointment letter. At the baseline clinic, written informed consent for all participants was taken by a trained research nurse before the collection of any data. All participants were made aware that they could withdraw from the study at any time. For each participant, a blood sample and clinical measurements were taken in accordance with local standard operating procedures, and a urine sample was collected. A hardcopy questionnaire booklet was completed at clinic by each participant. In addition, a hardcopy clinical research form was used to enter all other data collected at the baseline and 12-month time points, including demographic data, medical and medication histories, clinical measurements, blood results, and adverse events. Following clinics, all hardcopy study data collected were treated as confidential and kept securely in locked cabinets. Study data were anonymized and participants were identified by an allocated study number. These hardcopy data were then manually entered onto a validated, secure, and password-protected electronic database to be checked and verified prior to analysis.

### Randomization

Following confirmation of an eligible blood result for cholesterol, participants were randomly assigned at a 1:1 frequency by a trained member of the 3R team not involved with data collection, using an online randomization tool (Sealed Envelope Ltd, London, UK). Participants were stratified by age (40-53 years and 54-74 years) and sex before been randomly allocated to either the control or intervention group. If 2 people were taking part from the same household, they were automatically assigned to the same arm to prevent any contamination between groups taking place. We considered contamination between intervention and control participants from the same GP practice to be minimal based on evidence from a systematic review of contamination in trials of education interventions [24]. Although the intervention involved some fact giving, the main objective was motivation for behavior change and knowledge empowerment, which must be directly experienced to be effective and is therefore unlikely to result in contamination.

Following enrollment, blinding of participants was not possible due to the open and pragmatic design of the study. However, we took some steps to reduce bias: data were collected by research nurses not involved in any analysis; GPs were not informed of a participant’s group allocation; urine samples were analyzed by laboratory staff blinded to the randomized groups; and, prior to study completion, detailed protocol information was not made available online, which participants could have accessed.

### Control Group

The control group continued with their usual GP care with regard to lifestyle and medication advice for the primary prevention of CVD. To ensure that all participants had access to at least some basic knowledge about managing CVD risks, we sent both groups a British Heart Foundation booklet (*Keep Your Heart Healthy*), which contained general information about CVD risk prevention.

### Intervention Group

#### Development of the 3R Education Program and Follow-Up Support

We developed the 3R Education Program in line with UK Medical Research Council recommendations for the development of complex interventions [25,26]. We specifically focused on helping participants develop an increased sense of the role of their behaviors in their long-term health and risk by addressing their perceptions and beliefs surrounding CVD risk and medication adherence. In the absence of one unified model or construct, we used the concept of patient activation [27] and the capability, opportunity, and motivation model, as it encapsulates many theoretical components of behavior change theory [28]. We used the behavior change taxonomy [29] to ensure accurate reporting and to help identify the key active components of behavior change interventions.

An experienced working group of health professionals, led by a psychologist, developed the intervention based on the DESMOND philosophy of empowerment, whereby participants are supported, rather than taught, to discover and work out knowledge to achieve their own health goals [30]. We used several stages (including a literature search and focus groups with potential users) to explore and expand the original idea within the framework of the capability, opportunity, and motivation model [28]. This process informed the content, format, and theoretical basis of the program and was used to draft a curriculum to provide a written structure for the 3R Education Program and to identify the key component behavior change techniques to be used. We evaluated this using an iterative process of testing with potential users, feedback, and modification until we considered the program ready for implementation. We also developed educational, mixed-media resources to support the delivery of the curriculum and to engage participants in the learning activities. In conjunction with the development of the education program, a 2-day training program led by an experienced psychologist was delivered to a group of 6 facilitators to ensure consistent delivery of the curriculum. The newly trained facilitators were given the opportunity to practice delivery of the program before the start of the study.

In addition to the main education program, we developed follow-up support to help sustain any potential positive effects of the education with regard to improvements in medication adherence and other individual CVD risk factors. Continued support to maintain and promote positive health behavioral change has been shown to be effective when provided as brief telephone calls [31], and with SMS text messaging for medication adherence [32,33] and CVD risk factors [34,35]. After reviewing this literature, we considered the best format for the delivery of the 3R follow-up support to be a combination approach of using both text messages and phone calls to try to sustain the effect of the education.

For the text messages, this process involved using content that had already been robustly developed and validated in the Tobacco, Exercise and Diet Messages (TEXTME) study [36]. These text messages were originally developed for secondary CVD prevention but matched our requirements for primary CVD prevention: (1) general heart health information messages that included facts about CVD and information about medications and risk factors, (2) nutrition messages, (3) physical activity messages, and (4) smoking cessation messages. The message content had been specifically designed using behavioral change techniques, and a whole process of refining and piloting the messages had been applied. We had been impressed by the rigorous methods used to develop these messages and the success that they had achieved with the improvement of low-density lipoprotein cholesterol at 6 months, as well as improvement in other secondary end points (blood pressure, body mass index, smoking, and physical activity) [35]. Prior to use, the TEXTME messages were reviewed and adapted to make the content culturally appropriate for our UK patient cohort by 3 health professionals (a dietician, a nurse research fellow, and a GP). Furthermore, we created some additional medication reminder texts to be delivered in conjunction with the TEXTME texts that were specifically focused on improving medication adherence by prompting people to take their statin medication at a set time.

For the phone calls, we developed a semistructured script and a 1-day training session (involving practice role-play scenarios) using the same framework that we had used for the follow-up calls in the successful Pre‐diabetes Risk Education and Physical Activity Recommendation and Encouragement (PREPARE) study, which sustained the effect of a an educational intervention on glucose regulation at 2 years [31].

#### Delivery of the 3R Education Program and Follow-Up Support

We invited the intervention group to attend the 3R Education Program and receive the follow-up support (text messages and phone calls). They continued with their usual GP care and were sent a copy of the British Heart Foundation booklet. Textbox 1, Textbox 2, and Textbox 3 detail the different components involved in the delivery of each of the 3 major elements of the intervention and identify the behavioral control techniques [29] or active components involved in each.

### Outcomes and Measures

#### Primary Outcome

The primary outcome was medication adherence to statins at 12 months (Table 1) [37-44]. The primary measure was a urine-based biochemical measure involving a novel assay to test for statin and antihypertensive levels in urine samples. This method has already been used successfully to show poor adherence to antihypertensive medication [45]. Participants were informed that urine samples would be collected to assess the levels of statins and were asked to provide a first morning urine sample (in a standard urine collection tube) prior to clinic. This test uses a high-performance liquid chromatography–tandem mass spectrometry analysis of spot urine samples (2-5 mL) to detect 60 commonly prescribed cardiovascular medications, including statins. The results are reported as binary outcomes (yes or no) and hence are based on the limit of detection of a medication. The limit of detection is in the low nanomolar per liter range for all medications (in-house data). The method has been set up using established laboratory criteria for qualitative assays [46]. The method is robust and reliable and has been developed from techniques used in forensic medicine [47] and in testing for drug abuse in elite sports [48]. We use the test routinely in various cardiovascular clinics, and have set up the first National Centre for Drug Adherence Testing and receive samples from 25 centers across the United Kingdom [49].

**Ready to Reduce Risk (3R) Education Program.**

- Setting: local venue (eg, community hall).
- Format: group education (approximately 8 per group; participants were allowed to take a partner or friend) facilitated by a written structured curriculum and mixed-media educational resources (including a free pedometer).
- Frequency: 2 sessions.
- Duration: approximately 2 hours each.
- Facilitators: 2 trained facilitators (at least one was a health care professional).
- Training: 2-day course, self-study, and practice run.
- Monitoring: ad hoc filmed sessions.
- Outline
  - Session 1: explored understanding and beliefs to do with cardiovascular disease risk and how to manage it; showed how to calculate their own risk score using the Joint British Societies calculator; raised awareness of factors that influence risk, how these affect the body, and the role of medication; and explored beliefs about medication adherence.
  - Session 2: increased knowledge and awareness about how to have a healthier lifestyle to reduce cardiovascular disease risk and introduced behavioral control techniques to support activated participants.
- Theories and models: capability, opportunity, and motivation model; patient empowerment; working alliance; patient activation; self-regulation; self-determination; cognitive dissonance; self-efficacy.
- Behavioral control techniques: goal setting (outcome); problem solving; action planning; self-monitoring of outcome(s) of behavior; social support (emotional); social support (practical); information about health consequences; salience of consequences; demonstration of behavior; pros and cons; adding objects to the environment; incompatible beliefs; valued self-identity.

**Follow-up support phone calls after the Ready to Reduce Risk (3R) Education Program.**

- Setting: participants were called at home by a member of the 3R study team from a private office and designated study mobile.
- Format: participants received individual calls facilitated by a semistructured script and delivered using a patient-centered approach.
- Frequency: 2 calls at approximately 2 weeks and 6 months.
- Duration: approximately 10-20 minutes each.
- Facilitator: a trained member of the 3R team who was experienced in calling research participants.
- Monitoring: written records of the calls were documented by the facilitator, using a structured template.
- Outline: participants were called at a convenient time and asked some questions about how they were getting on following the 3R Education Program. The facilitator used open questions to elicit information about what had been going well and not so well, and participants were given the opportunity to discuss any pitfalls and ways to overcome these.
- Theories and models: the theoretical basis was the same as that for the 3R Education Program.
- Behavioral control techniques: goal setting (outcome); problem solving; action planning; self-monitoring of outcome(s) of behavior; social support (emotional); social support (practical); information about health consequences; pros and cons; valued self-identity.

**Text messaging follow-up support after the Ready to Reduce Risk (3R) Education Program.**

- Setting: 1 week posteducation, participants received text messages to their own mobiles via an independent text messaging service, which is set up to work from the secure study contacts database.
- Format: a series of automated, unidirectional text messages were sent, consisting of medication reminders (eg, “Have you taken your tablets today?”), and motivational and support Tobacco, Exercise and Diet Messages (TEXTME) messages (eg, “Walking up and down a flight of stairs several times is a great strengthening activity.”).
- Duration: 44 weeks*.*
- Frequency:
  - Medication reminders: weeks 1 and 2 (7 texts); weeks 3 and 4 (4 texts); weeks 5-26 (1 text); weeks 27 and 28 (7 texts); weeks 29 and 30 (4 texts); weeks 31-44 (1 texts). (Sent at the same time each evening.)
  - TEXTME messages: 4 texts per week. (Sent on random weekdays at random times.)
- Facilitator: texts were initiated and stopped manually by the 3R team.
- Training: participants received a *3R Follow-On Support* booklet and facilitators received training from their clinical research service (who developed the study database) on how to manage the text messaging support via the database interface.
- Monitoring: all texts sent were logged and monitored to identify any problems.
- Outline: participants could choose between a smokers’ and nonsmokers’ pathway for the type of texts that they received. A series of texts relating to healthy eating, physical activity, medication, general heart health, and smoking (if chosen) were then delivered as per the 44-week schedule detailed above. Texts could be stopped at any time by the participant, by sending a text to a specified number.
- Theories and models: the theoretical basis of the TEXTME messages is detailed in the protocol paper by Redfern et al [36].
- Behavioral control techniques: reduce prompts and cues (for medication reminders); other behavioral control techniques associated with the TEXTME messages are detailed in Redfern et al [36].

**Table 1:** Data collection schedule.

Demographic data		Measure	Baseline	12 months
Sex and age		Self-report	✓	N/A^a^
Medical history		Self-report	✓	✓
Medication history		Self-report/GP^b^ database	✓	✓
**Primary outcome**				
	Medication adherence to statins	Urine test	✓	✓
**Secondary outcomes**				
	Cardiovascular disease risk score (%)	QRISK2 calculator	✓	✓
	TC^c^, HDL^d^, TC:HDL (mmol/L)	Blood sample	✓	✓
	Blood pressure (mm Hg)	Omron monitor	✓	✓
	Body mass index (kg/m^2^)	Height (cm) and weight (kg)	✓ (height and weight)	✓ (weight only)
	Smoking history	Self-report using QRISK2 format	✓	✓
	Fruit and vegetable intake	Self-report: 5-a-day Community Evaluation Tool (FACET) [38]	✓	✓
	Quality of life	Self-report: 15D (a generic, comprehensive, 15-dimensional, standardized measure of health-related quality of life) [39]	✓	✓
	Health status	Self-report: EuroQol 5 dimensions questionnaire (EQ-5D, a standardized instrument for measuring generic health status) [40]	✓	✓
	Physical activity	Self-report: International Physical Activity Questionnaire (IPAQ; short form) [41]	✓	✓
	Patient activation	Self-report: Patient Activation Measure (PAM, a valid and reliable scale that reflects a developmental model of an individual’s readiness for health behavior change) [42]	✓	✓
	Medication/Health Beliefs	Self-report: Beliefs about Medicines Questionnaire (BMQ) [43] and the Brief Illness Perception Questionnaire (Brief IPQ) [44]	✓	✓
	Medication adherence to statins	Self-report: 8-item Morisky Medication Adherence Scale (MMAS)^f^ [37]	✓	✓
**Other supporting outcomes^e^**				
	Prescription history	Record of repeat prescription issues for statins and antihypertensives (if applicable) from the GP database	✓	✓
	Adverse events	Self-report and observed from GP database	✓	✓

^a^N/A: not applicable.

^b^GP: general practitioner.

^c^TC: total cholesterol.

^d^HDL: high-density lipoprotein.

^e^For the duration of the study.

^f^Use of the MMAS is protected by US and International copyright laws. Permission for use is required. A license agreement is available from: Donald E Morisky, MMAS Research (MORISKY), 294 Lindura Court, Las Vegas, NV 89138-4632; dmorisky@gmail.com.

In addition, the self-reported 8-item Morisky Medication Adherence Scale (MMAS) was completed at baseline and 12 months. This is an established and validated scale that is commonly used to measure adherence [37,50,51]. At the end of the study, we asked GP practices to provide details for individual participants regarding both statin and antihypertensive (if applicable) prescription issued during the 12 months of the study (Table 1). These data will provide useful supporting information about the pattern of patient medication adherence behavior over the 12-month study period.

#### Secondary Outcomes

Secondary outcomes were adherence to antihypertensive medications and other anticipated potential effects of the intervention, including a change in overall CVD risk score measured by the QRISK2 calculator [52], as well as potential changes in the following individual CVD risk factors: total cholesterol, high-density lipoprotein, total cholesterol to high-density lipoprotein ratio, blood pressure, and body mass index. We also observed changes in behavior and lifestyle using validated questionnaire measures: smoking, physical activity, fruit and vegetable intake, patient activation level, well-being, health status, health and medication beliefs, and medication adherence to antihypertensives (for participants prescribed this treatment for high blood pressure). We collected all outcome measures at the baseline and 12-month clinics in line with standard operating procedures and good clinical practice guidelines (Table 1). In addition, we checked for and recorded adverse events at the final visit and whenever a participant was contacted, for the purpose of the study, to monitor health status during the course of the study. Table 1 details all outcome data collected at the specified time points.

#### Process Outcomes

We also collected process outcomes relating to participant acceptability and fidelity of the education intervention. We used feedback data from the evaluation forms given out following the end of the education sessions to assess participant acceptability in conjunction with retention rates for the 2 sessions. We videotaped 3 sessions, with the participants’ permission, to assess the fidelity of the delivered education sessions. Logs were kept to record the initiation, delivery, and any terminations of text messages, and all attempted follow-up support phone calls were recorded.

### Sample Size

The primary outcome measure was medication adherence to statins at 12 months. We based the sample size calculation on the percentage of nonadherers by using data from the Investigation of Text Message Reminders on Adherence to Cardiac Treatment (INTERACT) trial [32]. To detect a difference in the proportion of medication adherers (of 16 percentage points in the intervention group at 12 months compared with the usual-care control group—ie, 91% compared with 74%), we required 84 participants per group with 80% power and 5% significance. After allowing for a 20% dropout, we required 105 participants per group, making 210 participants in total. This minimum difference was based on a similar 16 percentage–point increase in adherence to cardiovascular preventive treatment observed in the INTERACT trial [32], which used text messaging as the sole intervention. This was a 6-month follow-up study with a final control group adherence of 75%. We envisaged similar adherence to medication at 12 months’ follow-up, following our complex intervention with continued follow-up support via SMS text messaging and phone calls to sustain any initial intervention effect.

The study design was pragmatic and reflected how it would be delivered if it were implemented in practice. The study was designed to mitigate the effects of unbalanced clustering in the intervention group by using a standardized curriculum, a small group of trained facilitators to deliver education and phone calls, a small number of venues, standardized text messages, and standardized phone calls. Moreover, we assessed process outcomes relating to fidelity to allow for clear reporting of any variation that occurred. This was in line with the Medical Research Council guidance for complex interventions [26].

### Statistical Methods

For evaluation, we will summarize baseline characteristics of the 2 groups with means, standard deviations, and medians and ranges for continuous variables; and counts and percentages for categorical variables. Logistic regression will assess the difference in medication adherence by group, adjusted for the stratification factors (sex and age) at 12 months. We will assess the primary outcome at the 5% level with 95% confidence intervals. The primary analysis at 12 months will be based on complete data. The analysis of the secondary outcomes will be conducted in a similar manner using the appropriate model type: logistic regression for binary outcomes, linear for continuous outcomes, and ordinal for ordinal outcomes.

We will carry out sensitivity analyses on an intention-to-treat basis and a per-protocol basis to examine robustness of conclusions for missing data and attendance of the program. To adhere to the intention-to-treat principle, we will impute missing outcome data by multiple imputation using the command MI in Stata (StataCorp LLC). We will also conduct analysis by adding the MMAS adherence data where urine adherence data are missing.

### Ethics and Dissemination

We will disseminate the results of this study via the usual scientific forums: peer-reviewed publications and presentations at international conferences. At the local level, key stakeholders will be informed of the findings. The study has been administered by the Leicester Diabetes Centre and is overseen by the UK National Institute for Health Research Collaboration for Leadership in Applied Health Research and Care East Midlands Scientific Committee.

Ethics approval was obtained from the NHS Health Research Authority East Midlands-Leicester South Research Ethics Committee (15/EM/0472) prior to the commencement of the study. The trial is registered with the ISRCTN registry (ISRCTN16863160).

## Results

We recruited participants from May 2015 to March 2017, and have enrolled and randomly assigned a total of 212 participants. We finished follow-up clinic data collection in April 2018 and will commence the analysis of the results once we have collected and verified all data.

## Discussion

### Summary

This study aims to identify a potentially clinically useful and effective educational intervention to improve medication adherence to cardiovascular medications for the primary prevention of CVD, to be delivered as an adjunct to primary care.

This protocol paper offers a complete and thorough description of the 3R intervention and study methodology used prior to analysis and evaluation. A recent systematic review, looking at interventions to improve adherence to statin medication, highlighted that multifaceted interventions had small, positive effects on adherence, but that more methodologically rigorous trials are needed [53]. The 3R study has followed SPIRIT and CONSORT-EHEALTH guidance [22,23] to ensure rigorous methods are used, and publication of a protocol paper, prior to evaluation, allows this complex intervention to be described in the robust manner that is now expected. Due to significant amendments and potential biasing of outcome data from participants accessing information about the intervention, publishing a protocol paper prior to trial completion introduced several potential pitfalls for the reporting of the study. This is now a recognized problem for complex intervention trials that can be avoided by publishing protocols after trial completion [54].

We developed the 3R educational intervention in line with the Medical Research Council guidelines for complex interventions [25,26]. Moreover, we used a taxonomy of behavior change techniques [29] to identify the key active components to ensure more precise reporting of the intervention and to aid future research in this field. Due to the complexity of the 3R intervention, there will have been unavoidable variations in how the intervention was delivered, such as the use of different facilitators and venue settings; however, we monitored all components of the intervention to ensure that, as far as possible, the intervention was delivered as per protocol. We addressed process outcomes relating to patient acceptability and feasibility of the intervention and will carry out a cost-effectiveness analysis if the study proves to be successful.

### Conclusions

Medication adherence is a challenging primary outcome to measure, as no “gold standard” measure exists. In the 3R study, we have addressed this challenge by using a new and novel biochemical urine test as the primary measure [45]. Although this is an objective measure, it is essentially a spot-check of medication adherence to statins. There is also a bias with this measure because, ethically, participants have to be informed that their urine is being tested for statins. Therefore, we have also used a self-reported validated questionnaire (MMAS) [37,50,51] and repeat prescription history as supporting outcome measures. However, despite the challenges of this type of research, if it is successful, we hope that the 3R Education Program can be implemented within the primary care framework to improve medication adherence to statins and other CVD medications, and to provide better support for GPs and people at risk of CVD.

### Protocol Amendments

We have made the following significant changes to the original protocol. First, we revised the original time windows for conducting follow-up phone calls, as these were too restrictive and not practical. Second, we changed the primary outcome measure to the objective urine-based measure from the MMAS self-reported questionnaire measure. We made this change following additional validation data for the urine measure, which was not available at the start of the study.

## Appendix

Multimedia Appendix 1 Checklist of recommended items to address in a clinical trial protocol.

Multimedia Appendix 2 CONSORT-EHEALTH checklist V 1.6.1.

Multimedia Appendix 3 Peer-review report from CLAHRC-EM.

## Abbreviations

3R: Ready to Reduce Risk
CONSORT-EHEALTH: Consolidated Standards of Reporting Trials of Electronic and Mobile Health Applications and Online Telehealth
CVD: cardiovascular disease
DESMOND: Diabetes Education and Self Management for Ongoing and Newly Diagnosed
GP: general practitioner
INTERACT: Investigation of Text Message Reminders on Adherence to Cardiac Treatment
MIQUEST: Morbidity Query Information Export Syntax
MMAS: 8-item Morisky Medication Adherence Scale
NHS: National Health Service
PREPARE: Pre‐diabetes Risk Education and Physical Activity Recommendation and Encouragement
SMS: short message service
SPIRIT: Standard Protocol Items: Recommendations for Interventional Trials
TEXTME: Tobacco, Exercise and Diet Messages
