# Corrigendum and Editorial Warning Regarding Use of the MMAS Scale (The Ready to Reduce Risk (3R) Study for a Group Educational Intervention With Telephone and Text Messaging Support to Improve Medication Adherence for the Primary Prevention of Cardiovascular Disease: Protocol for a Randomized Controlled Trial)

**DOI:** 10.2196/13831

**Published:** 2019-07-24

**Authors:** Jo L Byrne, Helen M Dallosso, Stephen Rogers, Laura J Gray, Ghazala Waheed, Prashanth Patel, Pankaj Gupta, Yvonne Doherty, Melanie Davies, Kamlesh Khunti

**Affiliations:** 1 Leicester Diabetes Centre University Hospitals of Leicester National Health Service Trust Leicester United Kingdom; 2 Department of Health Sciences University of Leicester Leicester United Kingdom; 3 Innovation and Research Unit Northamptonshire Healthcare Foundation Trust Northampton United Kingdom; 4 Diabetes Research Centre University of Leicester Leicester United Kingdom; 5 Department of Cardiovascular Sciences University of Leicester Leicester United Kingdom; 6 Department of Clinical Pathology and Metabolic Sciences University Hospitals of Leicester National Health Service Trust Leicester United Kingdom; 7 York Diabetes Centre York Teaching Hospital National Health Service Foundation Trust York United Kingdom

## Authors’ Corrigendum

The authors of “The Ready to Reduce Risk (3R) Study for a Group Educational Intervention With Telephone and Text Messaging Support to Improve Medication Adherence for the Primary Prevention of Cardiovascular Disease: Protocol for a Randomized Controlled Trial” [JMIR Res Protoc 2018;7(11):e11289] require corrections in relationship to the use of the Morisky Medication Adherence Scale (MMAS-8).

The following text was omitted from the Acknowledgements and as a footnote in Table 1 and should be added:

Use of the MMAS is protected by US and International copyright laws. Permission for use is required. A license agreement is available from: Donald E Morisky, MMAS Research (MORISKY), 294 Lindura Court, Las Vegas, NV 89138-4632; dmorisky@gmail.com.

Two additional references should be cited after reference 37 in the following sentences:

- In addition, the self-reported 8-item Morisky Medication Adherence Scale (MMAS) was completed at baseline and 12 months. This is an established and validated scale that is commonly used to measure adherence [37].
- Self-report: 8-item Morisky Medication Adherence Scale (MMAS) [37].
- Therefore, we have also used a self-reported validated questionnaire (MMAS) [37] and repeat prescription history as supporting outcome measures.

The references to be cited are:

- Berlowitz DR, Foy CG, Kazis LE, Bolin LP, Conroy MB, Fitzpatrick P, SPRINT Research Group. Effect of Intensive Blood-Pressure Treatment on Patient-Reported Outcomes. N Engl J Med 2017 Dec 24;377(8):733-744.
- Morisky DE, DiMatteo MR. Improving the measurement of self-reported medication nonadherence: Final response. Journal of Clinical Epidemiology 2011 Mar;64(3):262-263.

These have become references 50 and 51, respectively, and all subsequent references have been renumbered accordingly.

The correction will appear in the online version of the paper on the JMIR website on July 24, 2019, together with the publication of this correction notice. Because this was made after submission to PubMed, PubMed Central, and other full-text repositories, the corrected article also has been resubmitted to those repositories.

## Editorial Notice

Authors and journal had to publish this correction due to legal threats by Steven Trubow and Donald Morisky from the company MMAS Research LLC, the copyright holder of the instrument. This is unfortunately not an isolated case, as the developers of this scale are known to comb the literature and ask those who used the scale for research to pay for a retroactive license which may cost thousands or tens of thousands of dollars, and to add references to their work [1]. This is now the fourth correction JMIR has to publish related to studies using the MMAS instrument [2-4].

The Committee on Publication Ethics (COPE) has recently discussed the ethics of this type of behavior by copyright holders of scales (“holding authors to ransom in this way”) and recommends to emphasize “the fact that this is not good for the advancement of scientific knowledge or in the public interest” [5]. As open access and open science publisher we remind our authors of our policies and preference for public and free availability of research tools, including questionnaires [6]. We actively discourage use of instruments which are not available under a Creative Commons Attribution license, and encourage our authors to use or develop/validate new instruments. We continue with our special call for papers for short paper instruments or electronic tools licensed under Creative Commons or available under an Open Source license that can be used as a free alternative to measure medication adherence, and will waive the article submission fee for such development and validation papers.
