# Supplementary material for: The Ready to Reduce Risk (3R) Study for a Group Educational Intervention With Telephone and Text Messaging Support to Improve Medication Adherence for the Primary Prevention of Cardiovascular Disease: Protocol for a Randomized Controlled Trial
Source: JMIR Res Protoc. 2018 Nov 12;7(11):e11289. doi: 10.2196/11289 (PMC6256101; doi:10.2196/11289)
Supplement: Multimedia Appendix 3 [file resprot_v7i11e11289_app3.pdf]

Diabetes Research Centre
University of Leicester
Leicester General Hospital
Leicester

T: 0116 258 8969
E:clahrc@nottingham.ac.uk
www.clahrc-em.nihr.ac.uk

04 December 2015

Prof Kamlesh Khunti
Diabetes Research Centre

Dear Kamlesh

Phase 1 Project 8. CVD Risk Prevention (3Rs) Study. Preventing Chronic Disease Theme.
In attendance: Carol Akroyd, Helen Dallosso

The Committee thank the Team for their presentation at the CLAHRC EM Scientific Committee Review Meeting on 6th November 2015

Prior to the presentation, it was noted the study had been previously rejected by an ethics committee, and had changed its primary outcome.

The project team noted difficulties in getting individuals to attend focus groups who were on statins. It was noted the eligibility criteria was changed due to change in the primary outcome measure.

The project had been rejected by the ethics committee in June 2015. The main points for rejection were that the protocol included too many recruitment strategies and collection of data from pharmacies. Recruitment strategies had been simplified and data was not being collected from pharmacy. The amended protocol has been approved by the sponsor and was resubmitted for ethics committee 19 November 2015.

Regarding progress of the study, the team reported that educators were now trained and were practising delivery of sessions. A database has been developed by the CTU. The hope was to start recruitment January/February 2016. The study has a target recruitment of 210, and it was noted that recruitment depended on the sampling pool and response rate. It was noted that formal feedback would not be collected from patients attending education sessions.

The Committee queried whether the primary outcome on page 155 of the meeting documents was correct, and the project team confirmed it was. It was advised to revise the protocol to be clear which measure is being used. It was noted that various statements throughout the protocol were confusing, and stated that the primary outcome variable was ordinal, binary and continuous in different parts of the protocol. This needs harmonisation so that the same primary outcome was used throughout the protocol. The main problem is that the sample size calculation is based on a comparison between 2 proportions, which is not consistent with the description of the primary outcome as ‘ordinal’, and the analysis method, which is a ‘linear model’

The Committee asked how individual statin adherence measured in the urine related to metabolism, and how much of a problem it would be to find this out in an assay that is still being developed. It was suggested to perhaps take this back to the project team. The Committee queried whether there were peer reviewed publications regarding validation of using statins in the proposed urine test.

The Committee queried the rationale for changing the primary outcome from cholesterol to adherence, and it was suggested to revert back. Showing that training to improve adherence improved adherence to medication in the short term would not be convincing although maintenance of such adherence over time would be important. Other studies discussing the value of medication have sometimes found that patients decide to adopt non-medication approaches to improve outcomes rather than take medication. If patients did this, then the educational intervention might look as if it did not improve outcome even though the steps people took in their lifestyle lowered cholesterol. Therefore improvements in cholesterol were the desirable outcome rather than medication adherence per se. It was also suggested that adherence to medication with validation of urine analyses could remain as secondary outcomes. If medication adherence was used as the primary outcome, it would be important to show that it continued in the medium term not just after the education, but the opinion of the committee was that lowering of cholesterol was the more desirable outcome.

The Committee commended the Team on the excellent training resources. The team were asked if a ‘Plan B’ was in place in case the application is rejected again by the Ethics Committee. The team did not offer one but were encouraged to consider one.

The Committee agreed that the team should re-think the primary outcome and consider consistencies in analysis.

The CLAHRC EM SC wishes to be updated on further progress with the study and that its suggestion will be utilised by the study team.

Best wishes

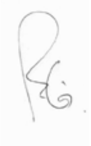

Paramjit Gill
CLAHRC EM Scientific Committee
